# Impact of TROP2 expression on prognosis in solid tumors: A Systematic Review and Meta-analysis

**DOI:** 10.1038/srep33658

**Published:** 2016-09-20

**Authors:** Ping Zeng, Min-Bin Chen, Li-Na Zhou, Min Tang, Chao-Ying Liu, Pei-Hua Lu

**Affiliations:** 1 Department of Radiotherapy and Oncology, Kunshan First People’s Hospital Affiliated to Jiangsu University, 91 Qianjin Road, Kunshan 215300, Jiangsu Province, China; 2 Department of Medical Oncology, Wuxi People’s Hospital of Nanjing Medical University, No. 299, Qingyang Road, Wuxi 214023, Jiangsu Province, China

## Abstract

Over-expression of TROP2 (the trophoblast cell surface antigen 2) was reported to predict poor prognosis in various solid tumors in number of studies. However, the results remained not comprehensive. Therefore, we here carried out this meta-analysis of relevant studies published on this topic to quantitatively evaluate the clinicopathological significance of TROP2 in solid tumors. Relevant articles were identified through searching the PubMed, Web of Science and Embase database. The primary outcomes were overall survival (OS) and disease-free survival (DFS). In this meta-analysis, 16 studies involving 2,569 participants were included, and we drew the conclusion that TROP2 overexpression was significantly associated with poor OS (pooled HR = 1.896, 95% CI = 1.599–2.247, *P* < 0.001) and short DFS (pooled HR = 2.336, 95% CI = 1.596–3.419, *P* < 0.001). Furthermore, the subgroup analysis revealed that the associations between TROP2 overexpression and the outcome endpoints (OS or DFS) were significant in in patients with female genital system neoplasms, as well in gastrointestine neoplasms. In addition, subgroup analysis found no difference HR across populations of different descent.Taken together, TROP2 overexpression was associated with poor survival in human solid tumors. TROP2 may be a valuable prognosis predictive biomarker and a potential therapeutic target in human solid tumors.

Trophoblast cell surface antigen 2 (TROP2), also known as GA733-1 or EGP-1 or TACSTD2, was a 36-kDa transmembrane glycoprotein, which was originally defined on normal and malignant trophoblast cells^1^,^2^,^3^. TROP2 has four potential N-linked glycosylation sites, and consists of 323 amino acids^4^. Trop-2 was synthesized in the endoplasmic reticulum, transported to, and glycosylated in the Golgi apparatus, and then sorted to the cell membrane^5^. Trop-2 was a calcium signal transducer that drives tumor growth and as quantitative tumor driver^3^,^5^. Existing evidences have shown that it plays a functional role in cancer progression and stem cells^6^,^7^. Altered expression and/or activity of TROP2 was involved in cancer cell growth, proliferation, migration, invasion and survival^8^,^9^,^10^,^11^,^12^,^13^. An increasing number of studies suggested that TROP2 is highly expressed in solid tumors, including gastric cancer^14^,^15^, nasopharyngeal carcinoma^16^, gallbladder cancer^17^, cervical cancer^18^, extranodal NK/T cell lymphoma^11^, lung cancer^9^,^19^, laryngeal squamous cell carcinoma^20^, colon cancer^21^,^22^,^23^, Hilar Cholangiocarcinoma^24^, pancreatic cancer^25^, squamous cell carcinoma of the oral cavity^26^, endometrioid endometrial carcinoma^27^, ovarian carcinoma^28^. These striking evidences on the role of TROP2 in cancer suggest the transmembrane glycoprotein would be further considered as a potential marker for outcome of cancer patients.

Many studies showed that increased TROP2 expression in tumor tissues was correlated with poor survival of patients with various cancer types. However, the results of those individual studies were not comprehensive. In the present study, we performed this comprehensive meta-analysis aiming to clarify the prognostic value of TROP2 in solid tumors and to support that the protein may be a potential therapeutic oncotarget.

## Materials and Methods

### Publication search

This present meta-analysis was performed under the Preferred Reporting Items for Systematics Reviews and Meta-Analyses guidelines^29^. A comprehensive literature search was performed using the electronic databases PubMed, Embase, and Web of Science databases (up to April 7, 2016) with the search terms: ‘TROP2’, ‘Trophoblast cell surface antigen 2′ and “cancer”/“tumor”/“neoplasm”/“carcinoma” and the following limits: Human, article in English or Chinese. All potentially eligible studies were retrieved and their bibliographies were carefully scanned to identify other eligible studies and extra studies were identified by a hand search of the references cited in the original studies. When multiple studies of the same patient population were identified, we included the published report with the largest sample size.

### Inclusion criteria

To be eligible for inclusion in this meta-analysis and data extraction, studies had to: (a) evaluation of TROP2 expression for predicting prognosis in cancer, (b) provide hazard ratios (HRs) with 95% confidence intervals (CIs) or enable calculation of these statistics from the data presented (c) classify TROP2 expression as “high” and “low” or “positive” and “negative”. (d) studies published in English.

### Exclusion criteria

Exclusion criteria were: (a) literatures published as letters, editorials, abstracts, reviews, case reports and expert opinions; (b) experiments performed *in vitro* or *in vivo*, but not based on patients; (c) articles without the HRs and 95% CI or K-M survival curves dealing with overall survival, disease-free survival; (d) The follow-up duration was shorter than 3 years.

### Data extraction

All data from the included studies were extracted independently and carefully by two reviewers using a standardized form. Disagreement was resolved through independently extracting data from the original article by the third author, and consensus was reached by discussions. The meta-analysis of TROP2 expression was based on two outcome endpoints: OS and DFS. Several different parameters, if reported, were extracted from each paper, including the first author’s surname, publication year, country of origin, number of patients analyzed, types of measurement, and score for TROP2 assessment, cut-off values to determine TROP2 overexpression, OS and DFS. The main features of these eligible studies are summarized in Table 1. OS was defined as the interval between initial diagnosis and death. DFS was the interval between initial diagnosis and recurrent or between disease progress and death. The multivariate HR was extracted to assess prognostic value of TROP2 expression. For the articles in which prognosis was plotted only as the Kaplan-Meier curves, the Engauge Digitizer V4.1 was then used to extract survival data, and the estimates of the HRs and 95% CIs were calculated by Tierney’s method^30^. All studies were assessed by Newcastle-Ottawa Scale (NOS). The quality scores ranged from 6 to 8, suggesting that the methodological quality was high.

### Statistical analysis

The data collected from each qualified paper of outcomes was used to evaluate the associations between TROP2 expression and solid cancer prognosis through meta-analysis. Pooled HRs and 95% CIs for two outcome endpoints (OS, DFS) were calculated. Subgroup analysis was performed when there were at least three studies in each subgroup. Statistical heterogeneity was assessed using the Q test, and a *P* value > 0.10 suggested a lack of heterogeneity among studies. We also quantified the effect of heterogeneity using *I*^2^ = 100% × (Q − df)/Q. *I*^2^ values of <25% may be considered “low”, values of about 50% may be considered “moderate” and values of >75% may be considered “high”^31^. According to the absence or presence of heterogeneity, random effects model or fixed effects model was used to merge the HR, respectively. Without statistical heterogeneity, a fixed effects model was employed to calculate the pooled HRs, otherwise random effects model was used^32^. Funnel plots and the Egger’s test were employed to estimate the possible publication bias^33^. If a publication bias did exist, its influence on the overall effect was assessed by the Duval and Tweedie’s trim and fill method^34^. Sensitivity analysis was also conducted to find out if certain single article could influence the overall result. Statistical analyses were conducted using Stata 14.0 (StataCorp, College Station, TX). P values for all comparisons were two-tailed and statistical significance was defined as p < 0.05 for all tests, except those for heterogeneity.

## Results

### Demographic characteristics

**Figure 1:**
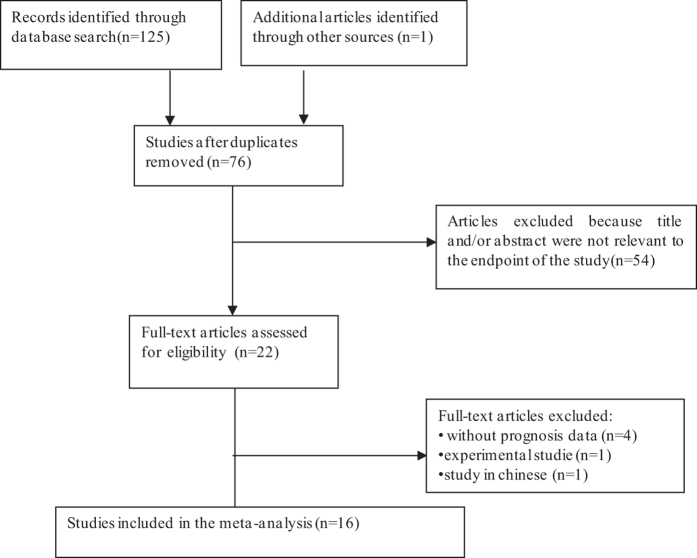
The flow chart of the selection process in our meta-analysis.

A total of 126 articles were retrieved by a literature search of the PubMed, Embase, and Web of Science databases, using different combinations of key terms. As showed in the search flow diagram (Fig. 1), 126 records were initially retrieved using the predefined search strategy. Because of repeated data, 50 records were removed. After browsing the retrieved titles and abstracts, 56 records were excluded due to no relevant endpoint provided. The remaining 22 records were downloaded as full-text and carefully accessed one by one. Among them, 6 studies were excluded, including one study that was experimental study, four that without prognosis data, one in chinese. As a result, 16 published studies including 2,569 patients that met the inclusion norm were finally selected for the meta-analysis, which assessed the relevance between TROP2 expression and solid tumor prognosis. The median sample-size was 97, with a wide range from 26 to 620. Among all cohorts, Mongoloid (n = 11) became the major race of literatures, followed by Caucasian (n = 5). As for the cancer type, twostudies evaluated gastric cancer, one study evaluated nasopharyngeal carcinoma, one study evaluated gallbladder cancer, one study evaluated cervical carcinoma, one study evaluated extranodal NK/T cell lymphoma, two studies evaluated lung cancer, one study evaluated laryngeal squamous cell carcinoma, one study evaluated Hilar Cholangiocarcinoma, two studies evaluated colorectal cancer, one study evaluated pancreatic cancer, one study evaluated squamous cell carcinoma of the oral cavity, one study evaluated endometrioid endometrial carcinoma, one study evaluated ovarian carcinoma. Overall, 15 studies focused on OS, 6 studies focused on DFS.

### Evidence synthesis

**Figure 2:**
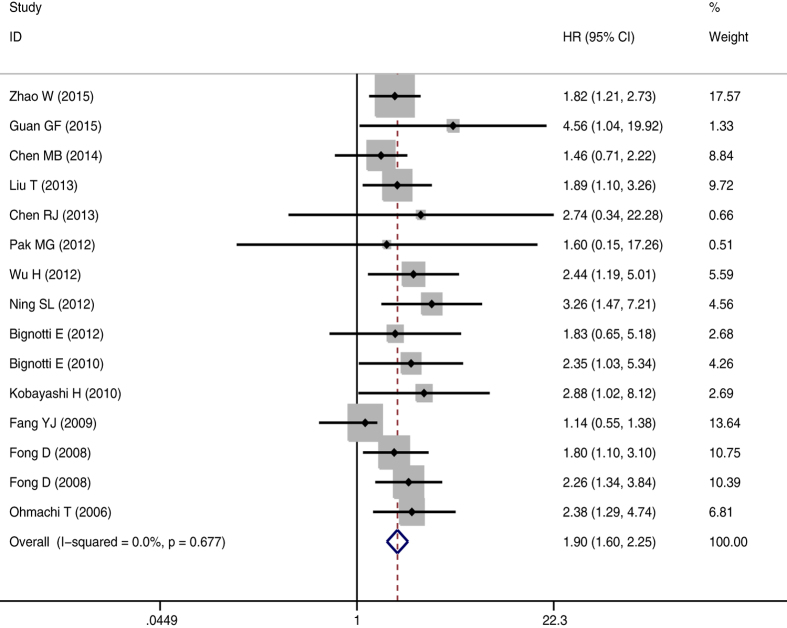
The correlation between TROP2 expression and overall survival (OS) in solid tumors.

**Figure 3:**
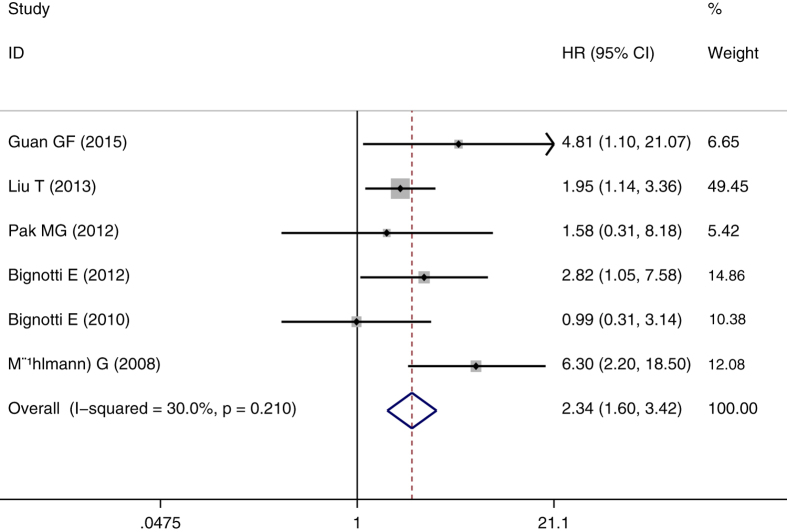
The correlation between TROP2 expression and disease-free survival (DFS) in solid tumors.

The meta-analysis of TROP2 expression was based on two outcome endpoints: OS, DFS. 15studies were included in the meta-analysis of OS. A fixed effects model was utilized to calculate the pooled hazard ratio (HR) and 95% confidence interval (CI) on account of the heterogeneity test reported a P value of 0.677 and an *I*^2^ values of 0.0%. The results suggested that TROP2 overexpression was associated with poor OS of solid tumors (pooled HR = 1.896, 95% CI = 1.599–2.247, *P* < 0.001) (Fig. 2). 6 studies were included in the meta-analysis of DFS. Owing to the heterogeneity test reported a P value of 0.210 and an *I*^2^ values of 30.0%, a fixed-effects model was used. The results show a significant association between TROP2 expression and DFS (pooled HR = 2.336, 95% CI = 1.596–3.419, *P* < 0.001) (Fig. 3). Subgroup study was then performed, the results suggested that the associations between TROP2 overexpression and poor OS and poor DFS were significant in Mongoloid patients (OS: pooled HR = 1.841, 95% CI 1.507–2.250, *P* < 0.001; DFS: pooled HR = 2.112, 95% CI = 1.299–3.433, *P* = 0.003), as well as in Caucasian (OS: pooled HR = 2.042, 95% CI 1.482–2.814, *P* < 0.001; DFS: pooled HR = 2.745, 95% CI 1.485–5.072, *P* = 0.001). The results showed that there was no difference OS or DFS HR across populations of different descent. The significant association was also detected between TROP2 overexpression and poor outcome in patients with female genital system neoplasms (OS: pooled HR = 1.989, 95% CI = 1.312–3.015, *P* = 0.001; DFS: pooled HR = 1.904, 95% CI = 1.227–2.954, *P* = 0.004) and gastrointestine neoplasms(OS: pooled HR = 1.642, 95% CI = 1.104–2.444, *P* = 0.014).

### Publication bias and sensitivity analysis

**Figure 4:**
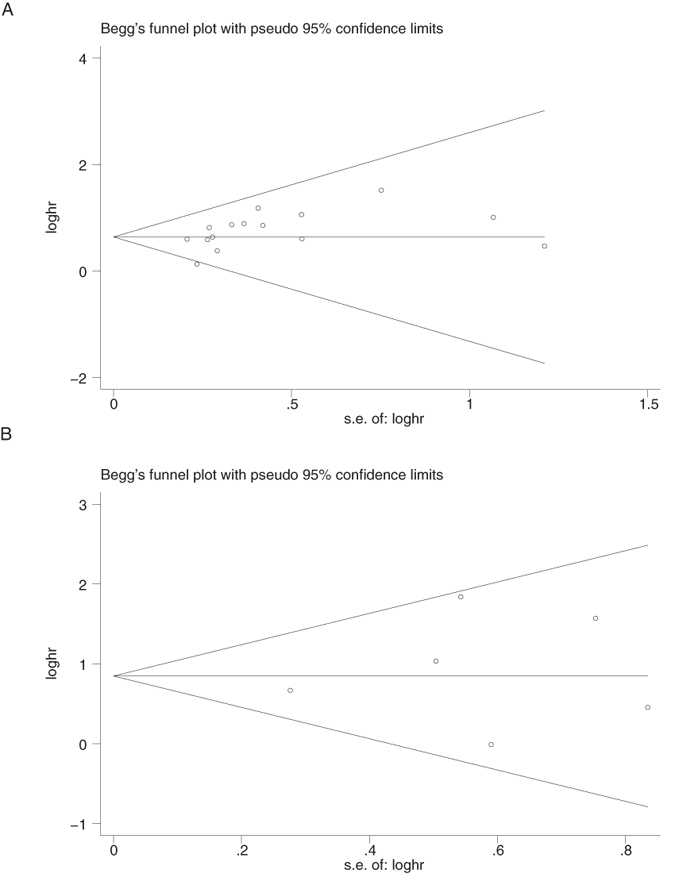
Begg’s funnel plots for the studies involved in the meta-analysis. (**A**) Overall survival. (**B**) disease-free survival(DFS). Abbreviations: loghr, logarithm of hazard ratios; s.e., standard error.

**Figure 5:**
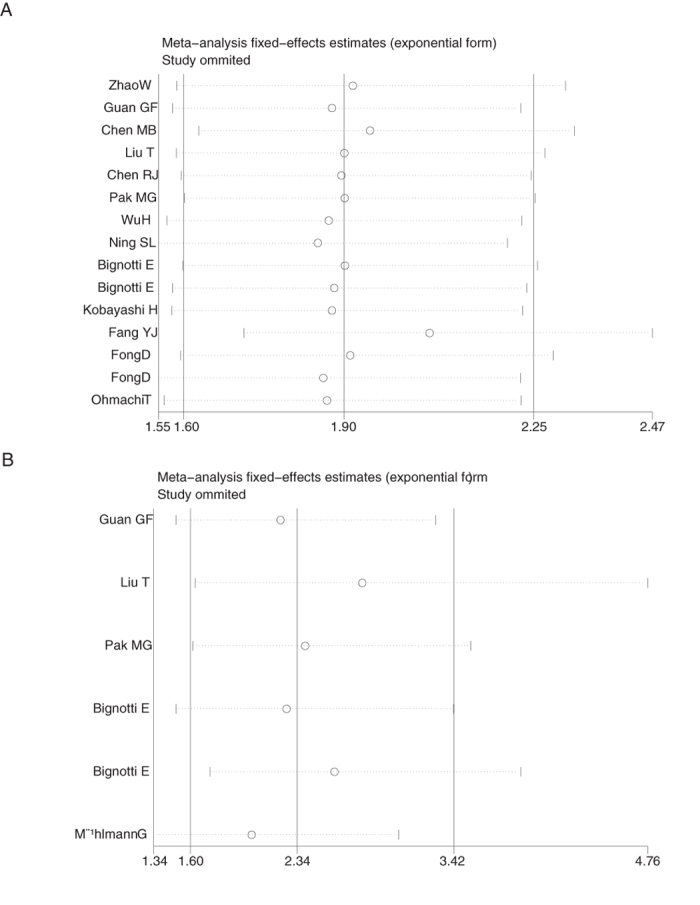
Sensitivity analysis of the meta-analysis. (**A**) Overall survival. (**B**) disease-free survival (DFS).

Begg’s funnel plot and Egger’s test were utilized to estimate the publication bias of the included literatures. The shapes of the funnel plots for the OS and DFS showed no evidence of obvious heterogeneity (Fig. 4), and Egger’s tests revealed publication bias concerning OS (P = 0.061) but not DFS (P = 0.653). Therefore, we performed trim and fill method to make pooled HR more reliable, and the P value was also <0.01(data not shown). Sensitivity analyses were further performed to determine the robustness of the results described above. No individual study dominated this meta-analysis, and the removal of any single study had no significant effect on the overall conclusion (Fig. 5).

## Discussions

High TROP2 expression had been reported to promote cancer progression and predict of poor prognosis of cancer patients^13^,^18^,^24^,^25^,^35^. Many clinical studies investigated the prognostic value of TROP2 over-expression. Most of these studies, however, include only limited numbers of patients and their conclusions remain not comprehensive. This current meta-analysis is the first complete overview of all reported clinical studies exploring the impact of TROP2 expression on prognosis of many solid tumors.

We systematically evaluated survival data of 2,569 solid tumor patients included in 16 different studies. Overall, these results clearly show that high TROP2 expression was a poor prognostic factor in solid tumors, with both results of poor OS (pooled HR = 1.896, 95% CI = 1.599–2.247, *P* < 0.001) and poor DFS (pooled HR = 2.336, 95% CI = 1.596–3.419, *P* < 0.001). Similarly, subgroup analysis revealed the associations between TROP2 overexpression and poor OS and DFS were significant within Mongoloid and Caucasian. When data was stratified according to cancer types, the results showed the prognostic value of TROP2 over-expression was significant in female genital system neoplasms and in gastrointestine neoplasms.

To our knowledge, the present study is the first and most full-scale meta-analysis systemically exploring the possible prognostic role of TROP2 up-regulation in solid tumors. Our quantitative results strongly supported the current mainstream viewpoint that an undesirable impact of TROP2 redundancy was correlated with the OS and DFS.

Additionally, several important implications in this meta-analysis were displayed. First, high TROP2 expression may be a general poor prognostic marker in solid tumors. In this meta-analysis, we included thirteen different cancer types, including gastric cancer^14^,^15^, nasopharyngeal carcinoma^16^, gallbladder cancer^17^, cervical cancer^18^, extranodal NK/T cell lymphoma^11^, lung cancer^9^,^19^, laryngeal squamous cell carcinoma^20^, Hilar Cholangiocarcinoma^24^, colon cancer^21^,^22^, pancreatic cancer^25^, squamous cell carcinoma of the oral cavity^26^, ovarian carcinoma^28^, endometrioid endometrial carcinoma^27^. The pooled results from these cancer types demonstrated that high TROP2 expression was associated with poor OS and DFS, and this finding can be extended to all solid tumors. Second, we demonstrated that high TROP2 expression correlated with poor OS and DFS in Mongoloid and Caucasian patients, as well in female genital system neoplasms and in gastrointestine neoplasms. Finally, it underlines the potential to develop TROP2 as a valuable therapeutic target and prognostic biomarker for solid tumors.

Apart from the inspiring outcomes, limitations still lay in this quantitative meta-analysis. First of all, most of the included studies were designed as retrospective studies, and such studies are more likely to be published if they have positive results than if they have negative results. Furthermore, the method for assessing TROP2 expression and definition of TROP2 positivity were inconsistent. Besides, some studies did not provide complete data^36^ or published in English^23^ were excluded in statistics. Therefore, our estimate of the associations between increased TROP2 and outcomes may have been overestimated.

In conclusion, high TROP2 expression in solid tumor tissues association with poor survival was clearly demonstrated in the present meta-analysis. We suggest that TROP2 may be a useful prognostic biomarker and a promising therapeutic target for solid tumors. Nevertheless, further studies related to specific tumor types and perspectives are required to corroborate the clinical utility of TROP2 expression in solid tumors.

## Additional Information

**How to cite this article**: Zeng, P. *et al*. Impact of TROP2 expression on prognosis in solid tumors: A Systematic Review and Meta-analysis. *Sci. Rep.* **6**, 33658; doi: 10.1038/srep33658 (2016).

## Figures and Tables

**Table 1:** Characteristics of studies included in the meta-analysis.

Author	Year	Country	Case	Disease	Method	Cut off value	Endpoints	NOS
Zhao W	2015	China	26	gastric cancer	IHC	score > 0(range of 0–300)	OS	6
Guan GF	2015	China	58	nasopharyngeal carcinoma	IHC	Low = score 0–1.5;high = score 2–3	OS, DFS	8
Chen MB	2014	china	70	gallbladder cancer	IHC	low (0–3) or high (4–9)	OS	8
Liu T	2013	China	74	Cervical Cancer	IHC	score > 0(range of 0–9)	OS, DFS	9
Chen RJ	2013	China	90	extranodal NK/T cell lymphoma, nasal type	IHC	HIS ≥ 4(range of 0–12)	OS	8
Pak MG	2012	South Korea	93	non-small cell lung cancer -adenocarcinoma	IHC	score > 4(range of 0–12)	OS,DFS	7
Wu H	2012	China	95	laryngeal squamous cell carcinoma	IHC	score > 0(range of 0–9)	OS	9
Ning SL	2012	China	97	Hilar Cholangiocarcinoma	IHC	score > 4(range of 0–12)	OS	9
Bignotti E	2012	Italy	100	endometrioid endometrial carcinoma	ICH	score = 3	OS,DFS	7
Bignotti E	2010	Italy	104	ovarian carcinoma	ICH	score = 3	OS,DFS	8
Kobayashi H	2010	Japan	106	pulmonary adenocarcinoma	IHC	score > 4(range of 0–12)	OS	8
Fang YJ	2009	China	109	colon cancer	IHC	immunoreactivity rating of II or III; moderate/strong	OS	9
Mühlmann G	2008	Austria	130	intestinal-type gastric cancer	IHC	score ≥ 4(range of 0–12)	DFS	9
Fong D	2008	Austria	197	pancreatic cancer	IHC	score ≥ 4(range of 0–12)	OS	7
Fong D	2008	Austria	600	squamous cell carcinoma of the oral cavity	IHC	score ≥ 4(range of 0–12)	OS	8
Ohmachi T	2006	Japan	620	Colorectal Cancer	RT-PCR	>95% of the expression values of the normal samples	OS	7

ICH:Immunohistochemistry.
